# Poisoning by Oil of Tansey

**Published:** 1833

**Authors:** 


### VI. Poisoning by Oil of Tansey.

Within the past year, two persons have been destroyed in this city, by the distilled oil of the *Tanacetum Vulgaris*. They were both young women. To the first it was given by mistake: The second, supposed by various circumstances to have taken it, appears to have done so for the purpose of committing suicide. We have not been able to learn the symptoms which preceded death in these cases; but mention the fact by way of inspiring caution, as the oil of tansey is hawked about our streets with many other essential oils, and sold to those who are ignorant of its deleterious properties. Linnæus observes, that the natural order, *Compositæ*, embraces no poisonous plants, except *Tagetes*, *Doronicum*, and *Armi-*

*ca.* It is probable, however, that many others, subjected to distillation, would, like the *Tanacetum*, yield a deleterious principle.

